# Associated factors and comorbidities in patients with pyoderma gangrenosum in Germany: a retrospective multicentric analysis in 259 patients

**DOI:** 10.1186/1750-1172-8-136

**Published:** 2013-09-08

**Authors:** Philipp Al Ghazal, Katharina Herberger, Jörg Schaller, Anke Strölin, Norman-Philipp Hoff, Tobias Goerge, Hannelore Roth, Eberhard Rabe, Sigrid Karrer, Regina Renner, Jan Maschke, Thomas Horn, Julia Hepp, Sabine Eming, Uwe Wollina, Markus Zutt, Isabell Sick, Benno Splieth, Dorothea Dill, Joachim Klode, Joachim Dissemond

**Affiliations:** 1 Department of Dermatology and Allergy, Skin Cancer Center Hannover, Hannover Medical School, Hannover, Germany; 2 Institute for Health Services Research in Dermatology and Nursing, University of Hamburg, Hamburg, Germany; 3 Department of Dermatology, St. Barbara Clinics, Duisburg, Germany; 4 Department of Dermatology, University of Tuebingen, Tuebingen, Germany; 5 Department of Dermatology, Heinrich Heine University, Duesseldorf, Germany; 6 Department of Dermatology, University of Muenster, Muenster, Germany; 7 Praxis für Venen- und Hauterkrankungen, Jena, Germany; 8 Department of Dermatology, University of Bonn, Bonn, Germany; 9 Department of Dermatology, Regensburg University Hospital, Regensburg, Germany; 10 Department of Dermatology, Venereology and Allergology, University Hospital of Leipzig, Leipzig, Germany; 11 Department of Dermatology, General Hospital of Goerlitz, Goerlitz, Germany; 12 Department of Dermatology, General Hospital of Krefeld, Krefeld, Germany; 13 Department of Dermatology and Allergology, University Hospital of Ulm, Ulm, Germany; 14 Department of Dermatology, University of Cologne, Cologne, Germany; 15 Hospital Dresden-Friedrichstadt, Academic Teaching Hospital of the Technical University of Dresden, Dresden, Germany; 16 Department of Dermatology and Allergology, Hospital of Bremen-Mitte, Bremen, Germany; 17 Department of Dermatology and Allergology, Ludwig-Maximilians-University Munich, Munich, Germany; 18 Departement of Dermatology, Vital Clinic, Alzenau, Germany; 19 Department of Dermatology, Hospital of Luedenscheid, Luedenscheid, Germany; 20 Department of Dermatology, Venereology and Allergology, University School of Medicine Essen-Duisburg, Hufelandstrasse 55, Essen 45122, Germany

**Keywords:** Pyoderma gangrenosum, Chronic ulcer, Comorbidities, Metabolic syndrome, Diabetes mellitus

## Abstract

**Background:**

Pyoderma gangrenosum (PG) is a rarely diagnosed ulcerative neutrophilic dermatosis with unknown origin that has been poorly characterized in clinical studies so far. Consequently there have been significant discussions about its associated factors and comorbidities. The aim of our multicenter study was to analyze current data from patients in dermatologic wound care centers in Germany in order to describe associated factors and comorbidities in patients with PG.

**Methods:**

Retrospective clinical investigation of patients with PG from dermatologic wound care centers in Germany.

**Results:**

We received data from 259 patients with PG from 20 different dermatologic wound care centers in Germany. Of these 142 (54.8%) patients were female, 117 (45.2%) were male; with an age range of 21 to 95 years, and a mean of 58 years. In our patient population we found 45.6% with anemia, 44.8% with endocrine diseases, 12.4% with internal malignancies, 9.3% with chronic inflammatory bowel diseases and 4.3% with elevated creatinine levels. Moreover 25.5% of all patients had a diabetes mellitus with some aspects of potential association with the metabolic syndrome.

**Conclusions:**

Our study describes one of the world’s largest populations with PG. Beside the well-known association with chronic bowel diseases and neoplasms, a potentially relevant new aspect is an association with endocrine diseases, in particular the metabolic syndrome, thyroid dysfunctions and renal disorders. Our findings represent clinically relevant new aspects. This may help to describe the patients’ characteristics and help to understand the underlying pathophysiology in these often misdiagnosed patients.

## Background

Pyoderma gangrenosum (PG) is a so far poorly characterized, challenging destructive neutrophil-mediated autoinflammatory disease with an incidence of 0.3-1.0/100.000 [1-5]. Langan et al. showed in 2012 an adjusted incidence rate standardized to European standard populations of 0.63 (95% confidence interval (CI) 0.57-0.71) per 100,000 person-years [6]. Even if the PG is a diagnosis of exclusion, for its proper identification various primary and secondary criteria should be fulfilled (Table 1). The clinical appearance is marked by the sudden onset of erythematous nodules or sterile pustules that rapidly develop into very painful ulcerations with violaceous undermined borders. Patients often report that primary lesions appear after trauma such as insect bites, excoriations or surgical interventions. The etiology of PG is still unclear. Clinical investigations have reported abnormal cellular immunity with anergy to recall antigens or an imbalance between helper T- and suppressor T-cells. Brooklyn et al. showed by the examination of T-cell receptor repertoire in cells taken from the peripheral blood and from PG biopsies that T-cells play an integral role in the development of PG and suggested that T-cells are trafficking to the skin under the influence of an antigenic stimulus [7]. An impairment of integrin effects on neutrophilic granulocytes, abnormal granulocyte tracking and monoclonal gammopathy involving IgA, IgG and IgM in up to 15% of patients has been discussed [8,9]. A currently investigated new aspect is the proline-rich, glutamic acid-rich, serine-rich and threonine-rich (PEST) family of protein tyrosine phosphatases as a critical regulator of cell adhesion and migration. The PSTPIP1 is a cytoskeleton-associated adaptor protein that links PEST-type phosphatases to their substrates. This pathway seems to be involved in diseases related to PG such as chronic inflammatory bowel disease and aseptic abscess syndrome [10,11]. Some case-reports about an autosomal dominant, auto-inflammatory disease, known as PAPA-syndrome (pyogenic arthritis, PG and acne) showed an association to mutations in the PSTPIP1/CD2BP1 gene on chromosome 15q. These mutations cause a hyper-phosphorylated PSTPIP1 protein and alter its participation in activation of the inflammasome with elevated interleukin-1 beta (IL-1β) release [12,13]. Demidowich et al. also investigated and confirmed these results by the analysis of five patients with PAPA syndrome and showed that mutations in PSTPIP1 are incompletely penetrant and variably expressed and neutrophil granule proteins were markedly elevated ex vivo and in the plasma [14]. The symptoms pyoderma gangrenosum, acne, and suppurative hidradenitis are summarized in the acronyme PASH syndrome as a new entity in the spectrum of autoimmunolatory syndromes. PASH syndrome is similar to PAPA syndrome but it differs in lacking the associated pyogenic arthritis. Mutations in the PSTPIP1 gens were excluded so far [15,16]. But in a currently published case report a p.E277D missense mutation of the PSTPIP1 gene was described as PAPASH syndrome [17].

**Table 1:** **Modified diagnostic criteria for PG**[1,18-23]

**I. main criteria**	Primary sterile pustule or ulcer with livid, undermined wound-border [1,14,15,18-21]
	Exclusion of other relevant differential diagnoses like chronic venous/arterial leg ulcer, pyodermatitis, vasculitis [1,14,15,18-20]
**II. additional criteria**	Histology of the wound-border: neutrophilic infiltration of the dermis with signs of vasculitis and accumulation of immunglobulins and/or complement factors beside the vessels [14,15,20]
	Existence of relevant, associated concomitant diseases like chronic inflammatory bowel diseases, arthropathies, haematological disorders, neoplasia, endocrine dysfunctions, metabolic syndrome [14,15]
	Response to a systemic immunosuppressive therapy or no response to *a* conventional ulcer-therapy [15,20]
	Triggering of a PG by pathergy-phenomenon [14,15,20]
	Extremely painful ulcer (VAS > 4 points) [14,15]

All these findings lead to a new effective targeted treatment with recombinant interleukin (IL)-1β receptor antagonists beside the already proven therapy with TNF-α inhibitors for blocking the key molecule of the inflammatory response [11].

Current knowledge about the pathogenesis of PG, as well as potential comorbidities is very limited and based on case series reports and results from studies with up to only 103 patients. Based on this information, some current textbooks report an association with internal diseases such as chronic inflammatory bowel disease, hematological disorders, neoplasia or other autoimmune diseases such as rheumatoid arthritis in up to 80% of patients [24]. The underlying original data are very heterogenous [1-3]. Therefore the aim of our multicenter study was to analyze current data from patients in dermatologic wound care centers in Germany in order to characterize associated factors and comorbidities in patients with PG.

## Material and methods

### Patients

This study was performed in compliance with the Helsinki Declaration by following ethical principles for medical research involving human subjects, including research on identifiable human material and data. After systematic literature analysis we developed a questionnaire and beginning in August 2010 advertised and recruited participation for this study among members of the working group for wound-healing (Arbeitsgemeinschaft Wundheilung, AGW) of the German Dermatology Society (Deutsche Dermatologische Gesellschaft, DDG). Data up to February 2011 were included in this analysis of patients with a diagnosed PG from 20 specialized dermatologic wound care centers in Germany. Positive written consent was obtained from each subject who participated in the study. The inclusion criteria for the patients in this investigation were the modified main and additional diagnostic criteria for PG as given in Table 1. After excluding clinically relevant differentials, the diagnosis has to be based in the centers on the patient medical history, histologic analysis of a biopsy sample, confirmation of the typical clinical appearance of PG lesions with a violaceous undermined border and the lack of response to conventional wound-therapy. The diagnoses were confirmed by selected experts from Essen (n = 49), Hamburg (n = 44), Duisburg (n = 29), Tübingen (n = 23), Düsseldorf (n = 20), Münster (n = 13), Jena (n = 12), Bonn (n = 10), Regensburg (n = 9), Leipzig (n = 9), Goerlitz (n = 7), Krefeld (n = 6), Ulm (n = 5), Cologne (n = 5), Dresden (n = 5), Göttingen (n = 5), Munich (n = 3), Alzenau (n = 3) and Lüdenscheid (n = 2) according to the best available diagnostic criteria. These diagnostic criteria are based on main criteria: presence of a primary sterile pustule or ulcer with livid, undermined wound-border, the exclusion of other differential diagnoses and at least one additional criterion (Table 1). Further studies will follow to validate and verify the clinical use of these proposed diagnostic criteria.

### Associated factors and comorbidities

According to the clinical records, concomitant diseases were classified in this questionnaire as gastrointestinal, rheumatoid, hematologic, or endocrine disease, as well as infectious disease, neoplasia, underlying immune deficiency and obesity. Obesity was defined as body mass index (BMI) >30.0 kg/m^2^. Pathologic serologic results and autoantibody detection were also recorded. Moreover, we collected demographic data such as gender, age, trigger factors and pain. Pain was measured by using a visual analogue scale (VAS) which ranged from 0 (no pain) to 10 (most severe pain imaginable).

### Statistical analysis

All data were recorded for each patient in an electronic table and statistically analyzed with the program Excel® from Microsoft® Office 2011.

## Results

### Patients

**Figure 1:**
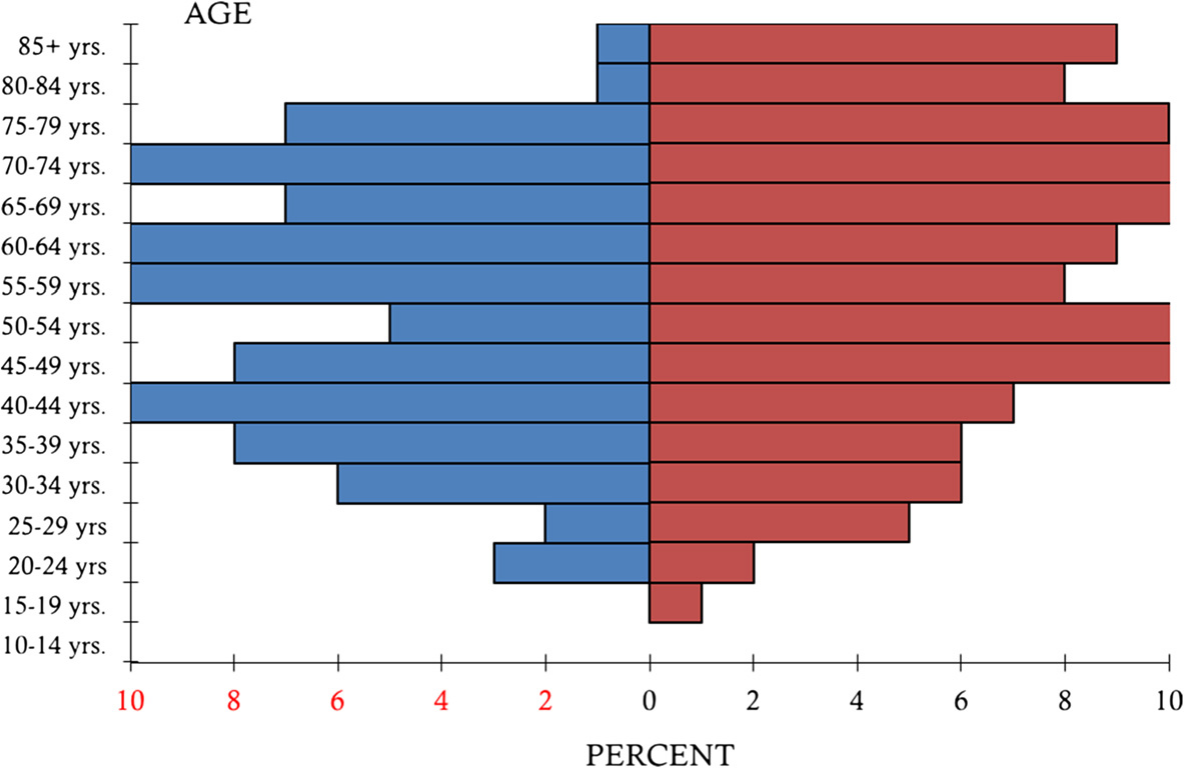
**Age pattern of male and female patients with PG.** Blue = male patients, red = female patients.

We received clinical data from 259 patients with PG from 20 different dermatologic wound care centers in Germany. Of these patients 142 (54.8%) were female, 117 (45.2%) were male. The age range for initial manifestation of PG was 21 to 95 years with a mean of 58 years. The average age of women was 60 years and that for men 54 years (Figure 1).

### Trigger

In 111 (42.8%) patients the onset of PG followed minor trauma or surgical intervention which was defined as a trigger. In 148 (57.2%) patients PG developed spontaneously without a relevant identified trigger.

### Pain

In this study patients described pain with a minimum of 2 points and a maximum of 10; the average score of the VAS scale was 6.2 when patients presented to the dermatologic wound care centers.

### Comorbidities

The comorbidities found in this study in patients with PG are summarized in Table 2. As a main finding of this investigation there is a potential association between endocrine diseases and the onset of PG. Diabetes mellitus was found in 25.5% of patients and thyroid disorders in 11.2%. As expected, inflammatory parameters were elevated in a large number of cases. Table 3 shows the comparison of our study data with the prevalence in median age groups of the several possible associated comorbidities.

**Table 2:** Comorbidities and pathologic serologic parameters in 259 patients with PG

	**Patients (absolute)**	**Patients (relative in%)**
**Gastrointestinal diseases**	**66**	**22.5 (overall)**
Chronic active hepatitis	11	16.7
Ulcerative colitis	17	25.8
Crohn’s disease	7	10.6
Diverticulosis	9	13.6
Gastric disorders	13	19.7
Gastric/duodenal ulcer	4	6.0
Gastrointestinal polyps	2	3.0
Steatohepatitis	1	1.5
Others	2	3.0
**Arthropathy**	**48**	**18.5 (overall)**
Rheumatoid arthritis	24	50.0
a) seropositive	21	43.8
b) seronegative	3	6.3
Arthrosis	17	25.8
Ankylosing spondylitis	2	4.1
Psoriasis arthropathy	3	6.3
Osteoporosis	2	4.1
**Haematological diseases**	**10**	**3.9 (overall)**
Leukemia	3	30.0
a) AML	1	10.0
b) CML	2	20.0
Lymphoma (T-cell)	2	20.0
Multiple myeloma	2	20.0
Macroglobulinemia	3	30.0
**Neoplasia**	**22**	**8.5 (overall)**
Breast cancer	6	27.3
Lung cancer	3	13.6
Prostate cancer	3	13.6
Malignant melanoma	3	13.6
Hepatocellular carcinoma	2	9.1
Ovarian cancer	2	9.1
Colorectal cancer	1	4.5
Laryngeal cancer	1	4.5
Glioblastoma	1	4.5
**Endocrine disorders**	**95**	**36.7 (overall)**
Diabetes mellitus	66	69.5
a) type II	60	63.2
b) type I	6	6.3
Thyroid disease	29	30.5
Hyperthyroidism	9	9.5
Hypothyroidism	18	19.0
Hashimoto’s thyroiditis	2	2.1
**Serological parameters**	**181**	**69.9 (overall)**
Anemia	118	65.2
Microcytic anaemia	28	15.5
Auto-antibodies	45	24.9
Anti-nuclear antibodies	30	16.6
C-reactive protein	181	100
Leukocytosis	117	64.6
Elevated creatinine	52	28.7
Renal failure	11	6.1

**Table 3:** Comparison of study data with prevalence in median age group according to current data from Germany

	**Our data**	**Prevalence in median age group**
Sex (m:f)	117:142; 55% female	prevalence for PG: 0.3-1.0/100.000
	(Germany: 1000:1040; 51% female)	
Age (mean)	21-94 years (57.3 years)	
	(Germany, 2010: 44.3 years)	
**Associated inflammatory disorders**		
Arthrosis, non rheumatoid arthritis	17 (6.6%)	female (45–65 years): 33%
		male: (45–65 years): 25%
Rheumatoid arthritis	24 (9.3%)	female (45–64 years): 8.6%
		male (45–64 years): 5%
		overall: 0.5-0.8%
Chronic inflammatory bowel disease	Overall: 24 (9.3%)	Crohn’s disease:
	Crohn’s disease:	120-200/100.000
	7 (2.7%)	Ulcerative colitis:
	Ulcerative colitis: 17 (6.6%)	160-250/100.000
**Endocrine disorders**		
Thyroid disease	29 (11.2%)	overall: 5.5%
		female: 8.9%
		male: 1.8%
Diabetes mellitus	66 (25.5%)	international overall (20–79 years): 12%
		German overall: 8.9%
Neoplasia	32 (12.4%)	overall: 5-yr.-prevalence: 1.6%
		10-yr.-prevalence: 2.4%
**Solid neoplasms (overall)**	22 (8.5%)	
Breast cancer	6 (2.3%)	female (50–59 years): 1-year-prevalence: 0.2%
Lung cancer	3 (1.6%)	female: 50–59 years: 1-year-prevalence: 0.04%
		male: 50–59 years: 1-year-prevalence: 0.07%
Prostate cancer	3 (1.6%)	male overall: 1-year-prevalence: 0.1%
		male (60–69 years): 1-year- prevalence: 0.5%
Melanoma	3 (1.6%)	female (50–59 years): 1-year.-prevalence: 0.03%
		male (50–59 years): 1-year life-prevalence: 0.02%
Hepatocellular carcinoma	2 (0.8%)	overall: 7-8/100.000
Ovarian cancer	2 (0.8%)	female (50–59 years): 1-year life-prevalence: 0.03%
Colon cancer	1 (0.4%)	female (50–59 years): 1-year.-prevalence: 0.1%
		male (50–59 years): 1-year-prevalence:
Laryngeal cancer	1 (0.4%)	female: 1-year-prevalence: <0.01%
		male: 1-year-prevalence: <0.1%
Glioblastoma multiforme	1 (0.4%)	n.a.
**Hematologic/hematopoetic neoplasia**		Leukemia, female:1-year-prevalence: 0.01%
AML	1 (0.4%)	
CML	2 (0.8%)	Leukemia, male:
Other	7 (2.8%)	1-year-prevalence: 0.01%

## Discussion

In the last few years there is increasing evidence that PG is not merely an isolated skin disease but instead may be a cutaneous manifestation of a generalized inflammatory reaction, which is associated with other internal diseases. It has been discussed that, due to a chronic systemic Th1-mediated inflammatory reaction, there is an increased incidence of metabolic syndrome [18,19]. Especially in obese patients the systemic inflammation with serological findings of increased pro-inflammatory cytokines such as TNF-α and L-6 leads to TNF-α-induced insulin resistance and subsequently to a pre-diabetic state. The patients suffer also from artherosclerosis and increased incidence of cardiovascular events [18]. In psoriasis there has been a paradigm shift away from viewing the disorder as a purely cutaneous disease to a systemic “immune-mediated inflammatory disease” (IMID). This group of diseases, which is also referred to as TRECID (TNF-α related chronic inflammatory diseases), includes psoriasis as well as Crohn’s disease, rheumatoid arthritis, and in current discussions maybe also PG [25]. All of these diseases involve a chronic systemic inflammatory reaction and respond clinically to TNF-α-antibodies.

The underlying data for current textbook chapters about PG are case report series and very few scientific studies with small numbers of patients [1-3,18-21,26-28]. In a single center investigation from Germany, data from 44 patients with PG were analyzed [1]. The authors reported an association between the onset of PG and autoimmune disease such as ulcerative colitis or Crohn’s disease in 6.8% (n = 3), and rheumatoid arthritis in 11.4%, while 20.4% had a malignant internal neoplasia. Two other clinical studies were published in 1985 and 2000 and included 86 patients [2,3]. Powell et al. performed a retrospective analysis of patients at two dermatologic centers in the United States (Table 4). They found 52.3% of all patients had a potentially associated systemic disease; 32.5% had an autoimmune disorder, 11.6% had ulcerative colitis, 9.3% patients had Crohn’s disease, and 11.6% patients had rheumatoid arthritis. Neoplasms were reported in 15.1% of patients and included monoclonal gammopathy, myelodysplasia, Hodgkin’s lymphoma, POEMS syndrome and IgA-myeloma. In this study numerous other factors such as infectious gastrointestinal diseases, cutaneous inflammation, and rare coagulation disorders were also examined. One single center, retrospective study from 1985 reported that 77.9% of patients had associated systemic disease. Arthritis was diagnosed in 37%, chronic inflammatory bowel disease in 36% and neoplasia, including monoclonal gammopathy and polycythemia vera in 17% of all patients. Endocrine disorders like diabetes mellitus were reported in only 2% of the patients.

**Table 4:** **Comparative summarized data of patients from the so far largest published case studies for PG between 1985 to 2011**[1-3,18,19]

	**Powell et al. (1985)**	**von den Driesch (1997)**	**Bennett et al. (2000)**	**Al Ghazal et al. (2011)**	**Binus et al. (2011)**	**Our data**
Patients	86	44	86	49	103	259
Sex (m:w)	1:1	1:2.1	1:1.3	1:1.5	1:3.1	1:1.2
Age (mean)	7-71 yrs.	11-80 yrs.	2-83 yrs.	22-95 yrs.	22-88 yrs.	21-94 yrs.
	(n.r.)	(50.3 yrs.)	(48.4 yrs.)	(59.7 yrs.)	(51.6 yrs.)	(57.3 yrs.)
Pathogenesis - trauma/surgery	23 (27%)	17 (39%)	n.r.	16 (33%)	32 (31.1%)	111 (42.8%)
**Inflammatory disorders**						
Arthrosis, non rheumatoid arthritis	28 (33%)	n.r.	10 (12%)	1 (2%)	20 (19.4%)	17 (6.6%)
Rheumatoid arthritis	n.r.	5 (11%)	n.r.	3 (6%)	10 (9.7%)	24 (9.3%)
chronic inflammatory bowel disease	31 (36%)	6 (14%)	18 (21%)	3 (6%)	35 (34%)	24 (9.3%)
	Crohn: 14 (16%)	Crohn: 3 (7%)	Crohn: 8 (9%)	Crohn: 1 (2%)	Crohn: 17 (16.5%)	Crohn: 7 (2.7%)
	Ulcerat. col.: 17 (20%)	Ulcerat. col.: 3 (7%)	Ulcerat. col.: 10 (12%)	Ulcerat. col.: 2 (4%)	Ulcerat. col: 18 (17.5%)	Ulcerat. col.: 17 (6.6%)
**Endocrine disorders**						
Thyreoid disease	5 (6%)	n.r.	n.r.	7 (14%)	4 (3.9%)	29 (11.2%)
Diabetes mellitus	2 (2%)	n.r.	n.r.	14 (29%)	29 (28.2%)	66 (25.5%)
Neoplasia						
	16 (19%) solid: 5 (6%)	5 (11%) solid: 1 (2%)	8 (9%) solid: n.r.	16 (33%) solid: 6 (12%)	21 (20.4%) solid: n.r.	32 (12.4%) solid: 22 (8.5%)
	hematolog.: 11 (13%)	hematolog.: 4 (9%)	hematolog.: 8 (9%)	hematolog: 5 (10%)	hematolog.: 21 (20.4%)	hematolog.: 10 (3.9%)
solid neoplasms	Bladder: 1 (1%)	Glioblastoma mult.: 1 (2%)	n.r.	Breast: 2 (4%)	n.r.	Breast: 6 (2.3%)
	Colon: 2 (2%)			Prostate: 1 (2%)		Lung: 3 (1.2%)
	Prostate: 1 (1%)			Melanoma: 2 (4%)		Prostate: 3 (1.2%)
				Glioblastoma mult.: 1 (2%)		Melanoma: 3 (1.2%)
						Liver: 2 (0.8%)
						Ovary: 2 (0.8%)
						Colon: 1 (0.4%)
						Larynx: 1 (0.4%)
						Glioblastoma mult.: 1 (0.4%)
**Haematological/haematopoetic neoplasia**						
AML	1 (1%)	n.r.	n.r.	0	1 (0.97%)	1 (0.4%)
CLL	n.r.	1 (2%)	n.r.	0	n.r.	0
CML	n.r.	1 (2%)	n.r.	2 (4%)	n.r.	2 (0.8%)
Other						
Myelodysplasia	n.r.	n.r.	3 (4%)	0	2 (1.9%)	0
Polycythemia rubra vera	1 (1%)	n.r.	n.r.	0	2 (1.9%)	0
Monoclonal gammopathy	9 (10%)	n.r.	4 (5%)	2 (4%)	10 (9.7%)	3 (1.2%)
Plasmocytoma	n.r.	1 (2%)		0	1 (1.0%)	2 (0.8%)
POEMS syndrome	n.r.	n.r.	1 (1%)	0	n.r.	0
Mycosis fungoides/ cutaneous T-cell lymphoma	n.r.	1 (2%)	n.r.	1 (2%)	n.r.	2 (0.8%)
Hodgkin’s lymphoma	n.r.	n.r.	n.r.	0	1 (1.0%)	0
Myelofibrosis	n.r.	n.r.	n.r.	0	1 (1.0%)	0
Large granular lymphocytic leukaemia	n.r.	n.r.	n.r.	0	1 (1.0%)	0
other Non-Hodgkin’s lymphoma	n.r.	n.r.	n.r.	0	1 (1.0%)	0

n. r. = not reported.

In our own single center investigation in 2011 we retrospectively analyzed data from 49 patients with PG from our dermatologic wound care center. Our results showed that only 6% of patients had chronic inflammatory bowel disease, 22.4% patients had a malignancy, 18.4% had elevated creatinine levels and 42.8% had anemia. A potentially relevant aspect that has received little attention so far was an association with endocrine disease in 38.8% of patients. Moreover 28.6% of our patients had diabetes mellitus. Given that 32.6% of patients were obese, we considered a potential association with metabolic syndrome as a possible new risk factor for PG [19].

The largest clinical trial to date was published by Binus et al. with 103 patients in 2011 [18]. Patient data were taken from the research patient data repository of a General Hospital in Massachusetts from 2000 to 2007 and retrospectively analyzed. In this study 34% of patients had an inflammatory bowel disease, 29.1% had arthritis, and 20.4% had a hematologic disorder, including monoclonal gammopathy of undetermined significance (MGUS, 9.7%) and hematologic malignancies (10.7%) like myelodysplastic syndrome, polycythemia vera, Hodgkin’s lymphoma, myelofibrosis, acute myelocytic leukaemia, large granular lymphocytic leukemia, multiple myeloma, and other Non-Hodgkin’s lymphoma. An underlying diabetes mellitus was found in 28.2% of all patients. Another 3.9% of all patients were diagnosed with chronic Hashimoto thyroiditis. Only 25.2% of the patients in this collective had none of the common comorbidities. The outcome of this study was an overall higher occurrence of associated illnesses in women (risk ratio = 1.2:1).

Another retrospective cohort study in 2012 by Langan et al. determined incidence, mortality of PG and the strength of the to date reported possible associations by analyzing a large, representative UK database [6]. Patients with pyoderma gangrenosum and 3 separate groups of age-, sex-, and practice-matched controls of the general population, patients with rheumatoid arthritis (RA), and patients with inflammatory bowel disease (IBD) were included in the study. In all there were 313 people with a median age of 59 years. The risk of death in their results was three times higher than that for general population controls (adjusted hazard ratio (ahr) = 3.03, 95% CI 1.84-4.73, p < 0.001), 72% higher than that for IBD controls (ahr = 1.72, 95% CI 1.17-2.59, p = 0.013), with a borderline increase compared to RA controls (adjusted hazard ratio = 1.55, 95% CI 1.01-2.37, p = 0.045). Disease associations were present in 33% of all participants: IBD, n = 67 (20.2%); rheumatoid arthritis n = 39 (11.8%) and hematologic disorders, n = 13 (3.9%). These results are consistent with our findings with regard to the underlying comorbidities even though the presence of IBD was less in our data.

In contrast to earlier studies, we found that only 9.3% of patients had chronic inflammatory bowel disease or rheumatoid arthritis (Table 4) [1-3,6,18,19]. The reasons for these differences are not clear. The advantage of a multicenter study however is that possible bias is likely less than in a single center study.

An important confirmation of previously published data was the association with hematologic neoplasia. In contrast to the results from Bolognia et al., who reported an association in up to 25% with neoplasms, 8.5% of our patients had an internal neoplasm and another 3.9% of the patients had a history of hematologic neoplasia, bringing the total to 12.4% of patients with potentially associated neoplasia [29]. As a new aspect we observed a potential association between endocrine diseases and PG. Diabetes mellitus, mostly type II, was found in 25.5% of patients and hypothyroidism in 6.9%. In this study 28.6% of patients had diabetes mellitus.

Our results also showed a potential association with elevated creatinine levels in 20.1% of the patients, and renal failure in 4.3% of our patients. Anemia was detected in 45.6% of patients (Table 2). This is another, as yet unpublished, possible cofactor in the pathogenesis of PG. The rates reported for diabetes mellitus vary between the different clinical studies. This may be the result of studies being performed in different countries, at different times and thus with different patient populations. Another factor is that in retrospective analyses only those data may be analyzed which were initially collected and reported. Until now association between PG and diabetes mellitus or metabolic syndrome has only been suggested by Binus et al. in 2011 and in our own study in 2012 [18,19]. Because of methodical problems in a retrospective analysis it is possible that there is a considerable underreporting and that the actual rates may be much higher.

### Strengths and limitations

One limitation of this multicenter, retrospective study is the question whether the diagnosis of a PG was correct in each case, because up to now there exist no worldwide accepted and standardized diagnostic criteria. Therefore every investigator of this study is an expert in dermatology, worked in a leading position in a dermatologic wound care center and had long lasting experience in the diagnosis of immunologic skin ulcers, which may reduce these errors.

Nevertheless, selection bias cannot be fully excluded due to our recruitment limited exclusively to wound care centers in dermatologic departments. In Germany PG is an integral part of dermatologic diagnoses. Therefore, if a wound is suspected as a PG, nearly all patients - independent of wound size or other factors - are referred to a dermatologic wound care center. The participating wound care centers in this study do not represent all of Germany’s wound care centers. However we consider the data to reflect a representative cross section of the PG population in Germany. The different number of reported patients from the several centers is based on methodological aspects and does not automatically correlate to total number of patients with PG treated in recent years in those centers. Another limitation is that for some patients the obtained medical history was incomplete. Therefore we can not exclude that at least some of the described associated comorbidities are the result of prior treatment of the PG. Moreover it is well known that a retrospective study based on a questionnaire can never include all relevant factors as well as a prospective investigation.

## Conclusions

This study, with a total amount of 259 patients with PG, represents the one of the largest studies of potentially associated factors and comorbidities in PG worldwide. Our data demonstrate a possible association with endocrine disorders, in particular the metabolic syndrome, which should be taken into consideration in future diagnostic and therapeutic investigations and protocols. Even though current data on the relationship between wound healing disorders and metabolic syndrome are scant, further studies are desirable, especially on the relationship between serologic concentrations of inflammatory mediators such as adipokines and wound healing [30,31]. Furthermore we observed until now not described possible associations with anaemia and renal dysfunction. These new data can be the base for standardized guidelines for the diagnosis and treatment of patients with PG in the future.

### Consent

Written informed consent was obtained from the patient for the publication of this report and any accompanying images.

## Abbreviations

AGW: Working group for wound-healing (Arbeitsgemeinschaft Wundheilung); AHR: Adjusted hazard ratio; BMI: Body mass index; CI: Confidence interval; DDG: German dermatology society (Deutsche Dermatologische Gesellschaft); IBD: Inflammatory bowel disease; IL: Interleukin; IMID: Immune-mediated inflammatory disease; N. R: Not reported; PAPA: Pyogenic arthritis pyoderma gangrenosum and acne; PASH: Pyoderma gangrenosum acne and suppurative hidradenitis; PAPASH: Pyogenic arthritis pyoderma gangrenosum acne and hidradenitis suppurativa; PEST: Proline-rich glutamic acid-rich serine-rich and threonine-rich sequence; PG: Pyoderma gangrenosum; RA: Rheumatoid arthritis; TNF: Tumor necrosis factor; TRECID: TNF-α related chronic inflammatory diseases; VAS: Visual analogue scale.

## Competing interests

The authors declare that they have no competing interests.

## Authors’ contributions

PA and JD made substantial contributions to conception and design, acquisition, statistical analysis and interpretation of data of this study and drafted the manuscript. KH, JS, AS, NPH, TG, HR, ER, SK, RR, JM, TH, JH, SE, UW, MZ, IS, BS, DD and JK have been sufficiently involved and participated in data acquisition, drafting the manuscript, interpretation of data and revising it critically for important intellectual content. All authors read the version to be published and have given final approval of the final manuscript to be published.
